# Hyperbaric oxygen therapy in children with post-concussion syndrome improves cognitive and behavioral function: a randomized controlled trial

**DOI:** 10.1038/s41598-022-19395-y

**Published:** 2022-09-23

**Authors:** Amir Hadanny, Merav Catalogna, Slava Yaniv, Orit Stolar, Lynn Rothstein, Adi Shabi, Gil Suzin, Efrat Sasson, Erez Lang, Shachar Finci, Nir Polak, Gregory Fishlev, Ruth Tock Harpaz, Moran Adler, Ron-El Goldman, Yonatan Zemel, Yair Bechor, Shai Efrati

**Affiliations:** 1 Sagol Center for Hyperbaric Medicine and Research, Shamir (Assaf Harofeh) Medical Center, Zerifin, Israel; 2 Sackler School of Medicine, Tel-Aviv University, Tel-Aviv, Israel; 3 Autism Center, Shamir (Assaf Harofeh) Medical Center, Zerifin, Israel; 4 Pediatric Neurology Department, Shamir (Assaf Harofeh) Medical Center, Zerifin, Israel; 5 Sagol School of Neuroscience, Tel-Aviv University, Tel-Aviv, Israel

**Keywords:** Brain injuries, Neurological manifestations, Brain injuries, Paediatric neurological disorders

## Abstract

Persistent post-concussion syndrome (PPCS) is a common and significant morbidity among children following traumatic brain injury (TBI) and the evidence for effective PPCS treatments remains limited. Recent studies have shown the beneficial effects of hyperbaric oxygen therapy (HBOT) in PPCS adult patients. This randomized, sham-control, double blind trial evaluated the effect of hyperbaric oxygen therapy (HBOT) on children (age 8–15) suffering from PPCS from mild-moderate TBI events six months to 10 years prior. Twenty-five children were randomized to receive 60 daily sessions of HBOT (n = 15) or sham (n = 10) treatments. Following HBOT, there was a significant increase in cognitive function including the general cognitive score (d = 0.598, p = 0.01), memory (d = 0.480, p = 0.02), executive function (d = 0.739, p = 0.003), PPCS symptoms including emotional score (p = 0.04, d = – 0.676), behavioral symptoms including hyperactivity (d = 0.244, p = 0.03), global executive composite score (d = 0.528, p = 0.001), planning/organizing score (d = 1.09, p = 0.007). Clinical outcomes correlated with significant improvements in brain MRI microstructural changes in the insula, supramarginal, lingual, inferior frontal and fusiform gyri. The study suggests that HBOT improves both cognitive and behavioral function, PPCS symptoms, and quality of life in pediatric PPCS patients at the chronic stage, even years after injury. Additional data is needed to optimize the protocol and to characterize the children who can benefit the most.

## Introduction

Persistent post-concussion syndrome (PPCS) is a common and significant morbidity among the pediatric population following traumatic brain injury (TBI)^1^. Mild TBI (mTBI) accounts for over 90% of all TBI in children, affecting more than 10% of children under the age of 16^2,3^. Although most of the children recover completely in a short period of time, about 10–30% suffer from PPCS, lasting from months to years^4^. PPCS has a significant effect on a child’s quality of life, school performance, family function and activities^5,6^. A recent survey confirmed that PPCS is underdiagnosed as over 25% of the children coming to the ED due to mTBI will suffer from PPCS^7^.

Guidelines for early management of acute concussion recommend limited periods of rest followed by a gradual return to school and other activities^8,9^. Early sub symptom threshold aerobic exercise has shown efficacy and speeds recovery in the first month post injury^10,11^. In contrast, the existing evidence for effective PPCS treatments remains limited^12^. Treatment usually include physical therapy, neurocognitive therapies among others. Graded exercise programs, cervicovestibular rehabilitation and multimodal collaborative care have been suggested in small studies^13–16^.

The beneficial effects of hyperbaric oxygen therapy (HBOT) has been recently shown in adults with chronic TBI and PPCS^17–21^. The combined action of hyperoxia and hyperbaric pressure, leads to significant improvements in tissue oxygenation, while targeting both oxygen and pressure sensitive genes^17^. Preclinical models and clinical studies in TBI survivors have demonstrated the effect of HBOT through several mechanisms including anti-inflammatory, mitochondrial function restoration, increased perfusion via angiogenesis and induction of proliferation and migration of stem cells^17,18,22,23^. Importantly, previous randomized controlled studies conducted on adults with PPCS showed that HBOT can be effective even years after injury^20,24^. However, the effects of HBOT on PPCS in the pediatric population has never been evaluated.

The aim of the study was to evaluate the effects of HBOT on a pediatric population suffering from PPCS after TBI, with ongoing symptoms for at least six months after injury, in a randomized, sham-control, double blind clinical trial.

## Results

### Patient characteristics and randomization

**Figure 1:**
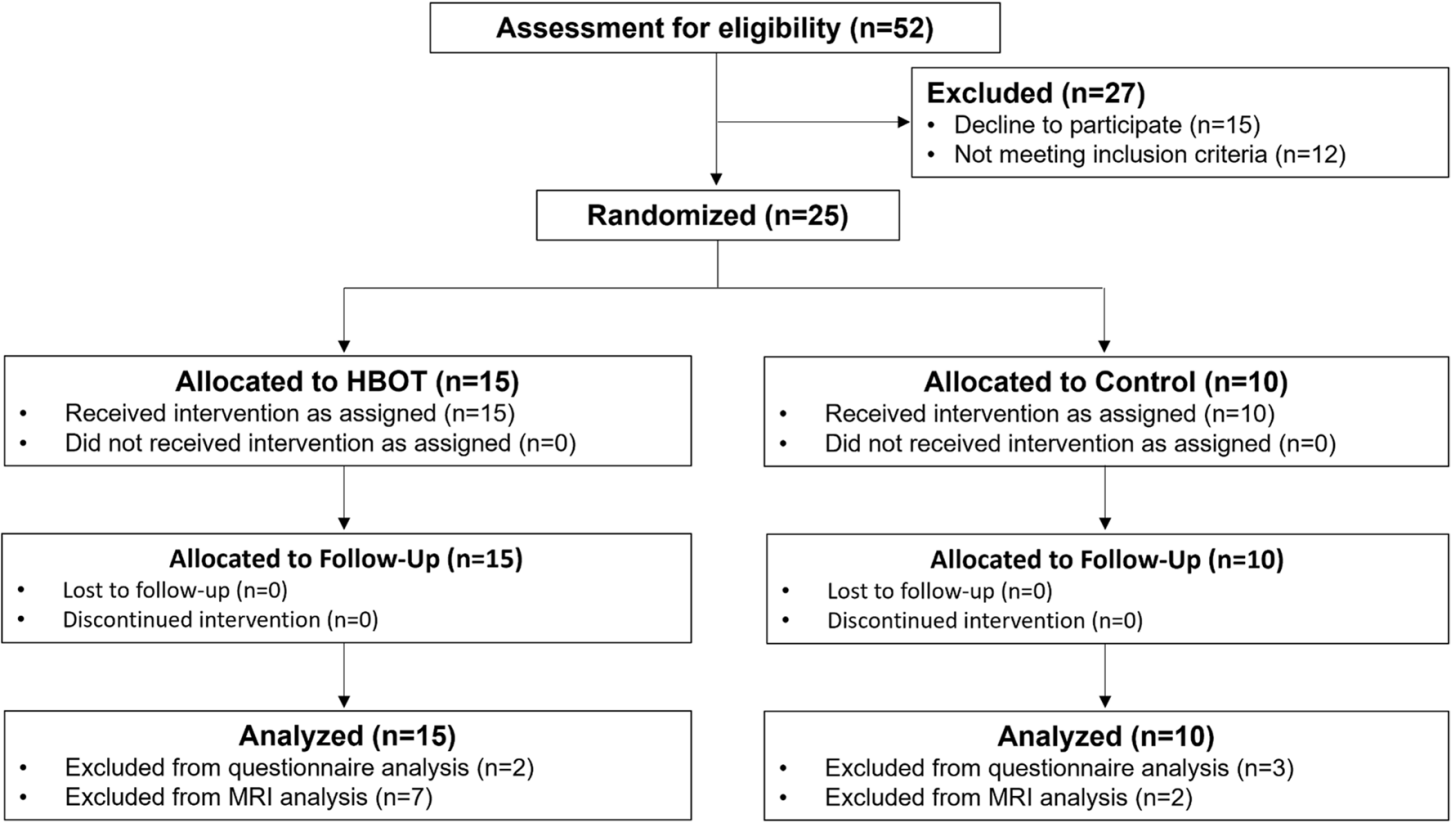
Patient flowchart.

Out of 52 children that were assessed for eligibility, 15 declined to participate, and 12 did not meet the screening inclusion criteria. Study recruitment was stopped prior to reaching the required sample size since parents of eligible children refused to participate in a sham controlled study. A total of 25 included children completed baseline assessments and randomized to either HBOT or sham arms. Eight children (four of the HBOT and four of the sham group) were under ten and did not complete the pretreatment Neurotrax cognitive evaluation. All other included children were over ten and completed all cognitive assessments (Fig. 1). The baseline characteristics of the cohort are provided in Table 1. The mean age of patients at inclusion and at injury were 11.6 ± 2.32 and 6.7 ± 3.18, respectively, and 80% were males. There were no significant differences in demographics, TBI characteristics, time from injury, or PPCS symptoms between the two arms.

**Table 1:** Baseline characteristics.

	HBOT group	Control group	P-value
N	15	10	
Age	11.99 ± 2.32	11.00 ± 2.32	0.308
Adolescents	7 (46.7)	3 (30.0)	0.678
Gender	12 (80.0)	8 (80.0)	1.000
Number of siblings	3.40 ± 2.75	3.00 ± 0.94	0.663
Age at injury	7.10 ± 3.52	6.15 ± 2.70	0.477
Time after injury	4.89 ± 2.23	4.85 ± 1.78	0.965
**Mechanism of injury**			
Motor vehicle collision	2 (13.3)	4 (40.0)	0.175
Bicycle accident	2 (13.3)	1 (10.0)	1.000
Fall	8 (53.3)	4 (40.0)	0.688
Struck by object/ blow	3 (20.0)	1 (10.0)	0.626
Arrival by EMS	12 (80.0)	7 (70.0)	0.653
Skull fractures	5 (33.3)	1 (10.0)	0.345
Abnormal imaging finding	5 (33.3)	4 (40.0)	1.000
Hospitalization	7 (46.7)	5 (50.0)	1.000
**Symptoms**			
LOC	5 (33.3)	2 (20.0)	0.659
Amnesia	3 (20.0)	3 (30.0)	0.653
Vomiting	6 (40.0)	3 (30.0)	0.691
**Severity score**			
Mild	11 (73.3)	7 (70.0)	1.000
Moderate	2 (13.3)	3 (30.0)	0.358
Severe	2 (13.3)	0 (0.0)	0.500
**Presence of persistent post-concussion symptoms**			
Learning difficulties	15 (100.0)	8 (80.0)	0.150
Poor concentration	14 (93.3)	8 (80.0)	0.543
Social difficulties	5 (33.3)	6 (60.0)	0.241
Forgetfulness/memory	8 (53.3)	7 (70.0)	0.678
Chronic headache	7 (46.7)	3 (30.0)	0.678
Depression/ anxiety	7 (46.7)	6 (60.0)	0.688
Current ADHD medications	4 (26.7)	3 (30.0)	1.000

Data presented as n (%); continuous data, mean ± SD.

Patient blinding was found to be reliable, where 11/15 (73.3%) of the HBOT group and 7/10 (70%) of the sham group perceived they were treated by HBOT (p = 1).

### Study endpoints

#### Cognitive function

**Figure 2:**
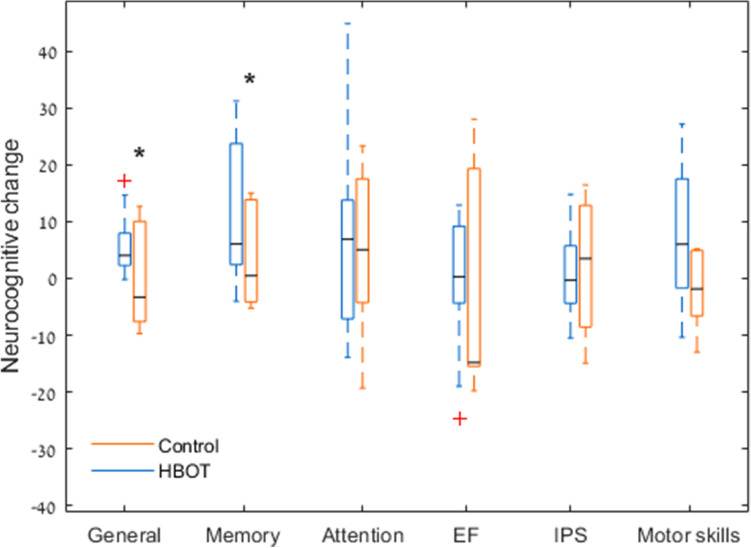
Primary endpoint changes boxplot. Both memory and the general cognitive scores were significantly increased after HBOT, with no changes in the sham group. Data shown as absolute mean change per cognitive domain. The central mark indicates the median, and the bottom and top edges of the box indicate the 25th and 75th percentiles, respectively. Red + symbols indicate outliers. *P < 0.05.

Results of the cognitive function evaluations are summarized in Table 2. The two groups had similar baseline scores in all cognitive domains, evaluated by the Neurotrax computerized battery. The general cognitive score was significantly improved in the HBOT (90.2 ± 10.3 to 96.1 ± 10.3, p = 0.01) compared to the sham group (95.1 ± 7.5 to 94.9 ± 7.7, p = 0.96), with a medium effect size of 0.598 (see Fig. 2). The most striking change was found in the memory domain with a medium effect size (d = 0.480) and a mean change of 11.5 ± 12.5 (p = 0.017) following HBOT, compared to 3.4 ± 8.8 (p = 0.39) (see Fig. 2). There were no statistically significant changes in other cognitive domains.

**Table 2:** Cognitive function changes.

	HBOT				Control				P-value baseline	Net effect size*
	Pre	Post	Change	Three months P-value	Pre	Post	Change	Three months P-value		
**Computerized tests**										
N	10				6					
General	90.2 ± 10.3	96.1 ± 10.3	5.9 ± 5.8	**0.010**	95.2 ± 7.6	95.0 ± 7.7	(− 0.20) ± 9.6	0.960	0.320	0.60
Memory	90.75 ± 19.9	102.3 ± 9.8	11.5 ± 12.5	**0.017**	93.0 ± 11.8	96.4 ± 14.8	3.4 ± 8.9	0.390	0.800	0.48
Executive function	93.2 ± 11.0	92.3 ± 12.2	(− 0.89) ± 12.5	0.830	92.8 ± 16.7	89.9 ± 11.2	(− 2.9) ± 20.8	0.740	0.960	0.13
Attention	88.5 ± 10.7	95.8 ± 15.0	7.3 ± 17.9	0.230	93.23 ± 15.9	97.8 ± 8.9	4.6 ± 15.4	0.500	0.490	0.20
Information processing speed	90.2 ± 9.9	90.6 ± 11.0	0.38 ± 7.5	0.880	84.6 ± 15.1	86.9 ± 13.9	2.1 ± 13.7	0.770	0.440	0.11
Motor skills	88.7 ± 21.1	96.2 ± 17.7	7.6 ± 12.2	0.080	103.0 ± 9.9	100.8 ± 12.1	(− 2.2) ± 7.0	0.470	0.140	0.59
**Pen and paper tests**										
N	15				10					
WISC 4 digit span	13.3 ± 2.9	15.8 ± 3.2	2.5 ± 2.3	**0.001**	13.0 ± 1.9	13.7 ± 2.2	0.7 ± 1.3	0.169	0.769	0.89
WISC 4 coding	43.7 ± 11.9	47.2 ± 11.7	3.5 ± 7.4	0.086	34.6 ± 10.3	35.9 ± 12.5	1.3 ± 9.1	0.672	0.081	0.27
WISC 4 symbol search	24.1 ± 6.3	25.7 ± 5.0	1.6 ± 4.1	0.155	17.1 ± 4.8	21.9 ± 6.7	4.8 ± 4.2	0.010	**0.012**	−0.76
WISC 4 cancellation	84.1 ± 17.0	93.7 ± 18.2	9.5 ± 14.2	**0.021**	74.2 ± 18.9	81.4 ± 15.7	7.2 ± 12.5	0.121	0.217	0.17
NEPSY—animal sorting	7.6 ± 2.1	7.1 ± 1.4	−0.5 ± 1.9	0.346	5.1 ± 2.7	7.3 ± 2.7	1.9 ± 2.4	0.064	**0.025**	−1.13
RAVLT total learning	42.5 ± 9.2	51.5 ± 8.2	9.1 ± 11.9	**0.010**	42.8 ± 11.8	48.1 ± 11.7	5.3 ± 8.5	0.097	0.946	0.35
RAVLT learning rate	7.3 ± 2.5	7.0 ± 1.9	−0.3 ± 2.9	0.728	6.1 ± 2.8	6.8 ± 1.8	0.7 ± 2.7	0.486	0.323	−0.33
Phonemic fluency	17.8 ± 7.3	22.8 ± 8.3	5.0 ± 8.1	**0.031**	17.9 ± 11.4	15.9 ± 7.6	−2.0 ± 9.7	0.553	0.983	0.81
Semantic fluency	34.4 ± 8.2	39.3 ± 7.4	4.9 ± 6.9	**0.016**	27.3 ± 5.9	35.0 ± 5.5	7.7 ± 9.2	0.038	**0.041**	−0.36
TOMAL2 abstract visual memory	17.9 ± 8.9	22.9 ± 9.4	5.0 ± 11.5	0.115	15.9 ± 7.7	16.3 ± 11.7	0.4 ± 9.2	0.888	0.601	0.43
TOMAL2 memory for location	11.1 ± 5.3	13.7 ± 6.6	2.5 ± 5.9	0.116	7.2 ± 3.9	9.6 ± 4.8	2.3 ± 4.2	0.138	0.078	0.04
TOMAL2 manual imitation	33.0 ± 10.3	33.3 ± 8.6	0.3 ± 12.4	0.935	26.6 ± 12.0	25.6 ± 9.7	−1.0 ± 13.0	0.823	0.195	0.10
Trail making test A (TMT)	35.9 ± 8.9	35.5 ± 9.6	−0.3 ± 8.9	0.887	55.9 ± 27.6	49.9 ± 23.8	−6.1 ± 34.0	0.608	**0.021**	0.26
Trail making test B (TMT)	134.2 ± 77.4	95.7 ± 39.9	−38.5 ± 58.3	**0.023**	174.2 ± 95.3	140.7 ± 76.2	−33.6 ± 71.1	0.194	0.294	−0.08
Five points (1 min)	12.3 ± 5.5	16.1 ± 4.8	3.7 ± 3.4	**0.001**	9.8 ± 4.2	11.4 ± 4.5	1.6 ± 1.1	0.003	0.283	0.75
Five points (2 min)	19.8 ± 8.5	23.2 ± 6.5	4.4 ± 4.8	**0.004**	15.4 ± 7.6	16.5 ± 6.7	1.1 ± 3.8	0.434	0.254	0.74

Baseline comparison of p-values test the null hypothesis of equal means of the two groups at the baseline using an unpaired t-test; three months comparison of p-values test the null hypothesis of equal means of each group pre-post intervention (HBOT/sham respectively) using a paired t-test; bold, *P* < 0.05, Net effect size is the subtraction of Cohen’s D effect size of the control group from the HBOT group Cohen’s D effect size; Neurotrax scores are normalized to age.

Compared to the sham group, there were significant changes in the WISC-IV digit span test (HBOT: 2.5 ± 2.3 vs sham: 0.7 ± 1.3, p = 0.17, d = 0.891) and cancellation test (HBOT: 9.5 ± 14.2, p = 0.02 vs sham: 7.2 ± 12.5, p = 0.12) following HBOT. There were significant differences in the baseline scores of the WISC-IV symbol search between the groups.

In the RAVLT test, there was a significant increase in the RAVLT total learning following HBOT (HBOT: 9.1 ± 11.9, p = 0.01 vs sham: 5.3 ± 8.5, p = 0.09) with no change in the learning rate.

Phonemic fluency was significantly improved in the HBOT group (5.0 ± 8.1, p = 0.031), compared to the sham group (− 2.0 ± 9.7, p = 0.55) with a large effect size of 0.806.

In the 5PT test, both groups improved significantly at the one-minute score (HBOT: 3.7 ± 3.4, p = 0.001, sham: 1.6 ± 1.1, p = 0.003). However, after two minutes, only the HBOT group had a significant change (HBOT 4.4 ± 4.8, p = 0.004, sham: 1.1 ± 3.8, p = 0.43) with a medium effect size of 0.739.

No significant differences were found in the TOMAL2 test for both groups. There were significant differences in the baseline scores in semantic fluency, NEPSY animal sorting and the trail making test A. Results of the cognitive function evaluations are summarized in Table 2.

#### PPCS symptoms

Compared to the sham group, the HBOT group had significant improvements in the HBI total score (7.9 ± 10.7, p = 0.02, d = − 0.171), cognitive score (12.6 ± 19.1, p = 0.035, d = 0.269) and somatic score (12.3 ± 18.5, p = 0.034, d = − 0.02). The HBI emotional and fatigue scores did not change (p > 0.05).

In the BC-PSI questionnaire, both groups had significant increases in their emotional score with a large negative effect size (HBOT: 6.9 ± 11.1, p = 0.04, sham: 15.7 ± 16.2, p = 0.04, d = − 0.676). The HBOT group had a significant increase in BC-PSI cognitive score (10.3 ± 11.4, p = 0.007) with a small negative effect size (d = − 0.17).

The quality of life questionnaire, PedsQL showed significant improvements in both the psychosocial health summary score (9.2 ± 13.2, p = 0.027, d = 0.167) and the school functioning (12.7 ± 17.5, p = 0.023, d = 0.217) following HBOT.

Results of PPCS symptoms questionnaires are summarized in Table 3.

**Table 3:** PPCS symptoms changes.

	HBOT				Control				P-value baseline	Net effect size*
	Pre	Post	Change	Three months P-value	Pre	Post	Change	Three months P-value		
N	13				7					
**HBI**										
Total HBI score	60.7 ± 16.2	68.6 ± 15.9	7.9 ± 10.7	**0.020**	58.2 ± 20.9	68.1 ± 17.2	9.9 ± 12.8	0.086	0.784	−0.17
Cognitive score	40.6 ± 22.2	53.2 ± 20.4	12.6 ± 19.1	**0.035**	35.3 ± 23.7	42.5 ± 16.8	7.1 ± 22.5	0.433	0.644	0.27
Somatic score	62.7 ± 30.7	74.9 ± 24.1	12.3 ± 18.5	**0.035**	71.4 ± 21.0	84.1 ± 15.3	12.7 ± 23.1	0.196	0.531	−0.02
Emotional score	70.9 ± 19.2	73.5 ± 17.3	2.6 ± 21.6	0.676	67.7 ± 27.1	74.1 ± 25.0	6.3 ± 14.9	0.303	0.774	−0.19
Behavior score	57.8 ± 25.8	64.7 ± 25.0	6.8 ± 17.8	0.190	51.9 ± 28.6	64.0 ± 25.9	12.2 ± 15.1	0.077	0.657	−0.32
**BC-PSI**										
BC-PSI physical score	72.0 ± 26.6	81.9 ± 18.7	9.9 ± 17.9	0.069	68.4 ± 24.1	79.6 ± 12.9	11.2 ± 20.6	0.199	0.780	−0.07
BC-PSI cognitive score	64.1 ± 18.3	74.4 ± 15.5	10.3 ± 11.4	**0.007**	50.0 ± 27.5	63.1 ± 12.5	13.1 ± 24.0	0.199	0.210	−0.17
BC-PSI emotional score	71.5 ± 17.9	78.5 ± 16.1	6.9 ± 11.1	**0.044**	62.9 ± 24.9	78.6 ± 24.2	15.7 ± 16.2	**0.042**	0.406	−0.68
BC-PSI fatigue Score	60.6 ± 29.8	69.2 ± 24.3	8.7 ± 24.1	0.221	66.1 ± 22.9	82.1 ± 19.9	16.1 ± 22.5	0.108	0.691	−0.31
**PedsQL**										
Psychosocial health summary score	67.1 ± 13.3	76.3 ± 16.3	9.2 ± 13.2	**0.027**	56.4 ± 26.1	63.6 ± 21.5	7.1 ± 10.8	0.132	0.268	0.17
Physical functioning	77.4 ± 21.4	80.3 ± 20.8	2.9 ± 9.4	0.291	70.5 ± 20.2	79.0 ± 15.2	8.5 ± 10.5	0.076	0.517	−0.57
Emotional functioning	64.6 ± 18.0	72.7 ± 23.3	8.1 ± 23.5	0.239	60.7 ± 30.4	67.1 ± 31.6	6.4 ± 12.5	0.222	0.737	0.08
Social functioning	84.6 ± 13.7	91.5 ± 10.3	6.9 ± 14.1	0.102	69.3 ± 36.1	75.7 ± 34.3	6.4 ± 14.6	0.289	0.213	0.04
School functioning	51.9 ± 24.5	64.6 ± 27.3	12.7 ± 17.5	**0.023**	39.3 ± 21.8	47.9 ± 15.3	8.6 ± 21.7	0.337	0.293	0.22

Baseline comparison of p-values test the null hypothesis of equal means of the two groups at the baseline using an unpaired t-test; three months comparison of p-values test the null hypothesis of equal means of each group pre-post intervention (HBOT/sham respectively) using a paired t-test; bold, *P* < 0.05, net effect size is the subtraction of Cohen’s D effect size of the control group from the HBOT group Cohen’s D effect size.

#### Behavioral assessment

The Conners-3 parents questionnaire showed significant improvements with small to medium effect sizes post-HBOT in the following domains of the Conners 3 tool: hyperactivity score (− 6.6 ± 9.8, p = 0.031, d = 0.244), executive score (− 11.2 ± 11.6, p = 0.004, d = 0.153), and learning problem score (− 5.7 ± 9.2, p = 0.044, d = 0.59).

In the BRIEF questionnaire, following HBOT, there were significant changes in the global executive composite score (− 7.0 ± 6.2, p = 0.001, d = 0.528), planning/organizing score (− 7.7 ± 8.5, p = 0.007, d = 1.09), initiation (− 7.0 ± 7.1, p = 0.004, d = 0.781), inhibition score (− 8.4 ± 8.9, p = 0.005, d = 0.586), working memory (− 6.1 ± 9.3, p = 0.037, d = 0.258) and the metacognition index (− 6.4 ± 5.8, p = 0.002, d = 0.706) with medium to large effect sizes. There was a significant improvement in the shift parameter in the sham group with a small negative effect size (d = 0.428, p = 0.02). Both groups had significant improvement in the behavioral regulation (BR) index (d = − 0.202, p = 0.01).

**Figure 3:**
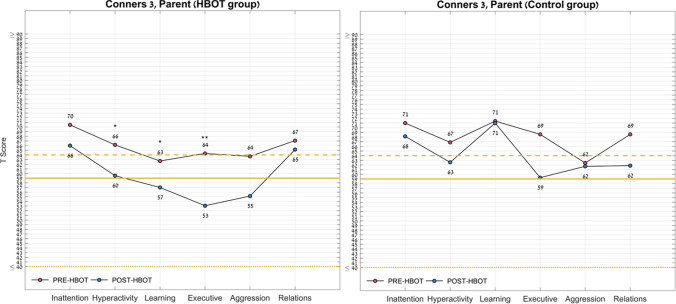
Conners scale for assessing ADHD questionnaire domains change. Following HBOT, the Conners 3 questionnaire showed significant improvements in the hyperactivity score, executive score, learning problem score and negative impression. Normal range [40–59]. *P < 0.05; **P < 0.01; See Table 4 for full statistical information.

**Figure 4:**
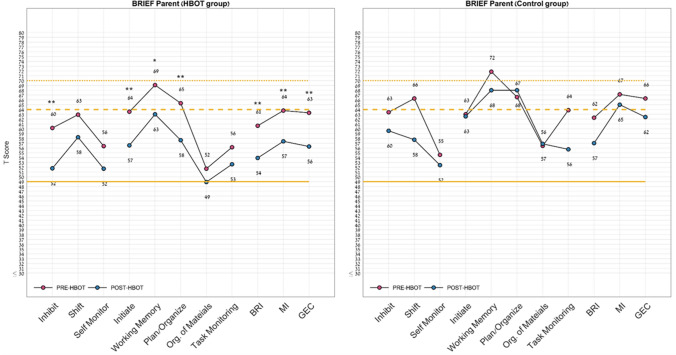
Behavior rating inventory of executive function questionnaire domains change. Following HBOT, the BRIEF questionnaire showed significant improvements in the inhibit, initiate, working memory, planning, behavioral regulation index, metacognition and global executive composite scores. Normal range, [49–64]; *BRIEF* behavior rating inventory of executive function. *P < 0.05; **P < 0.01; See Table 4 for full statistical information.

Results of the behavioral assessment data are summarized in Table 4 and Figs. 3 and 4.

**Table 4:** Behavioral symptoms changes.

	HBOT				Control				P-value baseline	Net effect size*
	Pre	Post	Change	Three months P-value	Pre	Post	Change	Three months P-value		
N	13				7					
**Conners 3**										
Inattention score	70.5 ± 15.5	66.0 ± 13.7	−4.5 ± 13.8	0.2666	71.0 ± 13.6	68.1 ± 11.6	−2.9 ± 5.9	0.2474	0.9424	−0.136
Hyperactivity score	66.2 ± 16.9	59.5 ± 12.2	−6.6 ± 9.8	**0.0319**	66.9 ± 18.6	62.6 ± 14.8	−4.3 ± 8.9	0.2498	0.9361	−0.244
Learning problems score	62.7 ± 12.3	57.0 ± 9.1	﻿−5.7 ± 9.2	**0.0446**	71.4 ± 15.6	71.0 ± 12.5	−0.4 ± 8.4	0.8976	0.2080	−0.59
Executive score	64.3 ± 13.8	53.1 ± 8.1	−11.2 ± 11.6	**0.0044**	68.6 ± 16.5	59.3 ± 11.7	−9.3 ± 14.8	0.1479	0.5675	−0.153
Aggression score	63.7 ± 19.1	55.2 ± 14.5	−8.5 ± 14.7	0.0577	62.4 ± 13.8	61.7 ± 17.3	−0.7 ± 9.4	0.8478	0.8848	−0.595
Peer relations score	67.1 ± 18.4	65.2 ± 17.2	−1.9 ± 9.9	0.4954	68.6 ± 17.2	61.9 ± 18.2	−6.7 ± 9.1	0.0973	0.8681	0.499
**BRIEF**										
Inhibit	60.2 ± 13.0	51.8 ± 10.2	−8.4 ± 8.9	**0.0052**	63.4 ± 17.4	59.6 ± 16.7	−3.9 ± 4.7	0.0732	0.6567	−0.586
Shift	62.9 ± 15.6	58.2 ± 13.1	−4.7 ± 9.9	0.1119	66.3 ± 14.5	57.7 ± 14.2	−8.6 ± 7.2	**0.0197**	0.6597	0.428
Self-monitor	56.4 ± 13.6	51.7 ± 11.9	−4.7 ± 7.8	**0.0504**	54.6 ± 13.7	52.4 ± 15.4	−2.1 ± 4.8	0.2863	0.7908	−0.367
Initiate	63.5 ± 10.4	56.5 ± 9.7	−7.0 ± 7.1	**0.0039**	63.0 ± 9.9	62.6 ± 13.4	−0.4 ± 10.6	0.9184	0.9164	−0.781
Working memory	69.1 ± 11.1	63.0 ± 10.4	−6.1 ± 9.3	**0.0371**	71.9 ± 9.0	68.0 ± 6.5	−3.9 ± 6.8	0.1864	0.5957	−0.258
Plan/organize	65.3 ± 13.9	57.6 ± 12.7	− 7.7 ± 8.5	**0.0067**	66.6 ± 9.1	68.0 ± 9.6	1.4 ± 8.0	0.6548	0.8396	−1.094
Or. of materials	51.7 ± 11.0	48.9 ± 9.5	− 2.8 ± 6.4	0.145	56.4 ± 11.5	56.9 ± 9.9	0.4 ± 6.1	0.8594	0.4043	−0.506
Task monitoring	56.2 ± 13.9	52.6 ± 11.1	− 3.5 ± 7.9	0.1342	63.9 ± 14.3	55.7 ± 14.0	− 8.1 ± 9.5	0.0637	0.2809	0.542
BRI	60.6 ± 14.8	53.9 ± 12.2	− 6.7 ± 7.8	**0.0093**	62.3 ± 17.0	57.0 ± 17.4	− 5.3 ± 4.9	**0.028**	0.8312	−0.202
MI	63.8 ± 12.2	57.4 ± 11.8	− 6.4 ± 5.8	**0.0019**	67.1 ± 9.4	65.0 ± 9.5	− 2.1 ± 6.4	0.409	0.5522	−0.706
GEC	63.3 ± 13.3	56.3 ± 12.1	− 7.0 ± 6.2	**0.0015**	66.3 ± 12.3	62.4 ± 12.5	− 3.9 ± 5.5	0.114	0.6475	−0.528

Baseline comparison of p-values test the null hypothesis of equal means of the two groups at baseline using an unpaired t-test; three-month comparison of p-values test the null hypothesis of equal means of each group pre-post intervention (HBOT/sham respectively) using a paired t-test; bold, *P* < 0.05, net effect size is the subtraction of Cohen’s D effect size of the control group from the HBOT group Cohen’s D effect size.

*BRI* behavioral regulation index, *MI* metacognition, *GEC* global executive composite.

#### Neuro-physical evaluation

The two groups had similar non-pathological baseline BESS scores (total score < 12, p > 0.05). Following HBOT, there was a positive trend in the firm surface total score (4.7 ± 3.0 to 3.4 ± 2.9, p = 0.054) with a large effect size of 0.99 compared to the sham group (4.2 ± 3.0 to 5.1 ± 2.9, p = 0.20). Results of the balance evaluations are summarized in Table 5.

**Table 5:** Neuro-physical changes.

	HBOT				Control				P-value baseline	Net effect size*
	Pre	Post	Change	Three months P-value	Pre	Post	Change	Three months P-value		
N	14				10					
FIRM	4.7 ± 3.0	3.4 ± 2.9	−1.3 ± 2.3	0.0537	4.2 ± 3.0	5.1 ± 2.9	0.9 ± 2.1	0.2042	0.6959	−0.997
FOAM	5.6 ± 3.2	6.1 ± 4.2	0.5 ± 5.8	0.7533	7.4 ± 5.0	7.3 ± 5.5	−0.1 ± 2.6	0.9074	0.3278	0.125
BESS (< 12)	10.4 ± 4.6	9.6 ± 7.0	−0.8 ± 6.8	0.6714	11.9 ± 8.2	11.6 ± 8.1	−0.3 ± 4.3	0.8321	0.5796	-0.082

Baseline comparison of p-values test the null hypothesis of equal means of the two groups at baseline using an unpaired t-test; three-month comparison of p-values test the null hypothesis of equal means of each group pre-post intervention (HBOT/sham respectively) using a paired t-test; bold, *P* < 0.05, net effect size is the subtraction of Cohen’s D effect size of the control group from the HBOT group Cohen’s D effect size.

#### Brain imaging

Nine patients were excluded due to having the pre- and post-scans in different MRI machines and head coils (due to replacement of the hospital’s MRI machine). A total of eight subjects of the HBOT group and eight of the sham group were included in the analysis.

**Figure 5:**
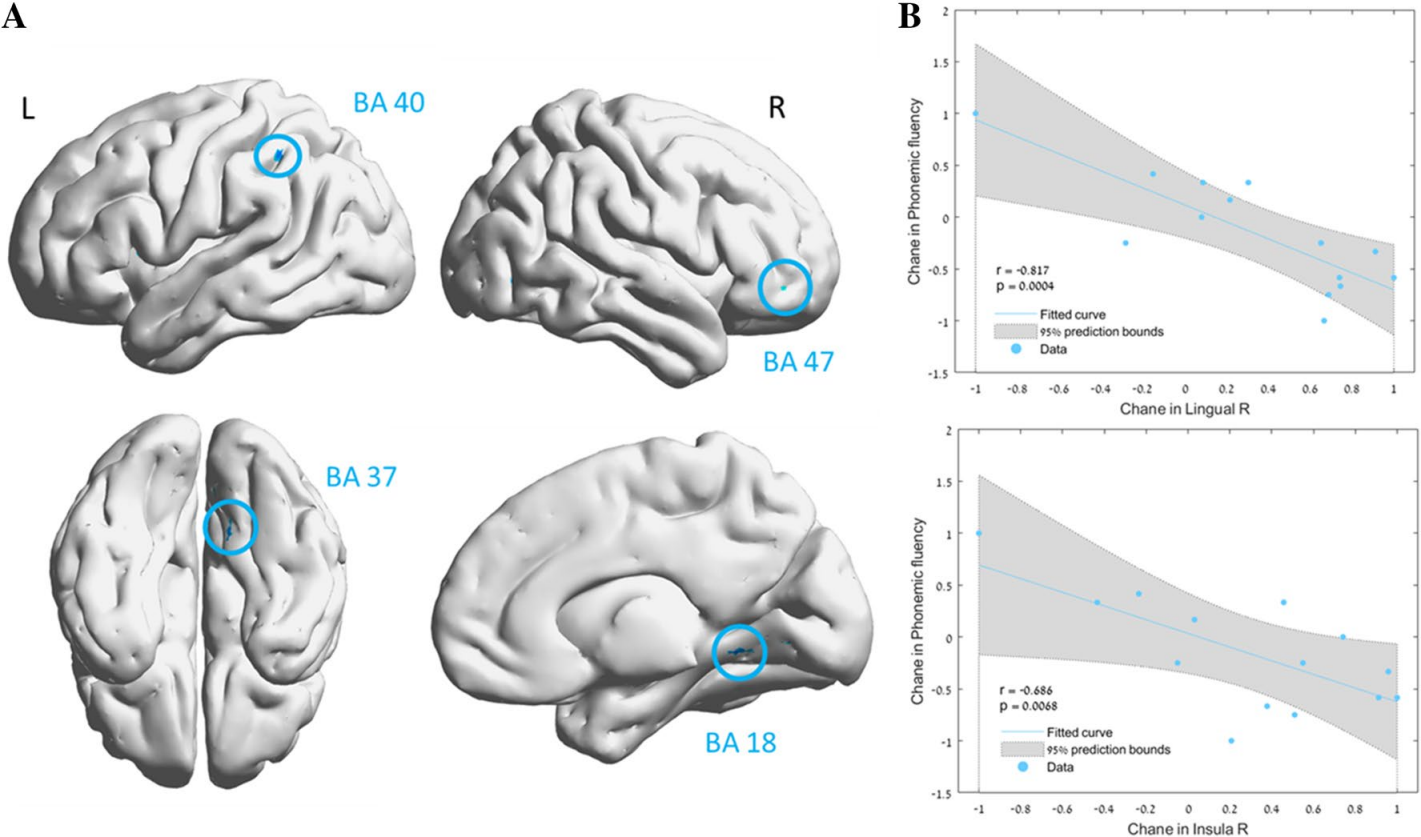
Brain regions with significant post HBOT changes in mean diffusivity (MD). Group-by-time interaction ANOVA model in: (**A**) DTI—mean diffusivity (MD) in gray matter, p < 0.005, uncorrected (**B**). Significant correlation between change in phonemic fluency and the right lingual MD (BA 18), and right insula MD (BA 13). r is Pearson's correlation coefficient. The 95% prediction interval is presented in the shaded area. *R* right, *L* left, *BA* Brodmann area. Brain images were created using BrainNet Viewer Software (http://www.nitrc.org/projects/bnv/).

Voxel-based analysis demonstrated significant mean diffusivity (MD) decreases in the HBOT group compared to the sham group in the following regions: right fusiform (BA37), right lingual gyri (BA18), right and left insula (BA13), left supramarginal gyrus (BA40), and the right and left inferior frontal gyri (BA47) (Table 6 and Fig. 5). There were strong correlations between MD changes and cognitive scores changes: phonemic fluency change correlated with MD changes in both the right lingual gyrus (r = − 0.817, p < 0.02), right and left insula (r = − 0.636–0.686, p < 0.0001) and left supramarginal gyrus (r = − 0.611, p = − 0.02.). The right insula MD changes correlated with both the WISV-IV digit span (r = − 0.591, p = 0.026) and the 5PT (2 min) changes (r = − 0.540, p = 0.046).

**Table 6:** Brain regions with significant mean diffusivity (MD) changes.

Anatomical location	BA	MNI coordinates			t-value	Cluster size	P-value
		X	Y	Z			
Fusiform R	37	48	−66	−2	3.89	50	0.00028
Lingual R	18	11	−67	0	3.52	122	0.00073
Insula L	13	−42	2	7	3.03	44	0.00121
Supra marginal L	40	−51	−44	48	3.31	29	0.00128
Inferior frontal L	47	−42	23	0	3.31	76	0.00128
Insula R	13	40	4	−13	3.07	58	0.00238
Inferior frontal R	47	39	37	−14	2.96	23	0.00307

The table reports each brain region found significant in a time-by-group repeated measures ANOVA comparing the two groups (HBOT N = 8, sham N = 8). The results are in specific Montreal Neurological Institute (MNI) coordinates; *X* sagittal, *Y* coronal, *Z* axial, refer to MNI. *BA* Brodmann area. All coordinates emerged at a threshold of p < 0.005, uncorrected. Minimum cluster size: 20; *R* right, *L* left.

#### Safety

Nine out of the 10 (90%) sham patients and 13/15 (86.7%) patients from the HBOT group had side effects (p = 1). Five out of the nine (55.6%) sham patients and 2/13 (15.3%) of the HBOT group suffered from otalgia. Additionally, 4/9 (44.4%) sham patients and 9/13 (69.2%) HBOT patients had signs of mild-moderate barotrauma (p = 1). All cases were treated conservatively. However, due to repeated events, two patients (one HBOT, one sham) required tympanic tube insertion to continue protocol sessions. Lastly, two HBOT treated patients complained of headaches which were treated conservatively.

## Discussion

This is the first randomized sham-controlled trial evaluating HBOT’s effect on pediatric PPCS patients in a prospective manner. We found that HBOT induces significant improvements in cognitive function, PPCS, behavioral symptoms and quality of life. The main improved cognitive domains include memory (in both Neurotrax, WISC-IV, RAVLT and verbal fluency) and executive function (in 5PT and trail making). Behavioral symptom changes included executive functioning and attention difficulties. These findings were associated with brain microstructural changes in the frontal, temporal and the paralimbic regions associated with cognitive roles.

Following TBIs, children and adolescents experience changes in both cognitive and behavioral functioning. The most common cognitive function deficits are working memory, executive functioning and attention. These can significantly affect a child’s learning capabilities and quality of life^25–28^. Moreover, the attention and executive functioning deficits impact progress in academics and psychosocial development, and may have a negative influence on family functioning^29,30^. Working memory is a temporary, capacity-limited memory holding store. This memory is critical for executive function that matures during puberty, adolescence and early adulthood^27^. We found a significant increase in working memory in both computerized battery and traditional (pen and paper) based tests. Executive functioning is a term used to describe a variety of abilities allowing purposeful, goal-directed, problem-solving behavior, including behavioral regulation, planning and organizational skills, and self-monitoring. In our study, we found that the HBOT group significantly improved in both the objective 5PT test as well as in the parents’ subjective scores in the BRIEF questionnaire.

Behavioral symptoms of attention difficulties are observed as inattention, hyperactivity and impulsivity, which are the primary symptoms associated with attention deficit hyperactivity disorder (ADHD)^31^. These symptoms are overrepresented in children with TBI, with 20% of children meeting ADHD criteria prior to the injury and an additional 20% of children experiencing new attention difficulties by two years post-injury^32^. Interestingly, no changes in attention measures were seen in cognitive tests in both groups. However, we found a significant improvement in all behavioral symptoms of attention difficulties in the Conners 3 questionnaire.

In order to evaluate intervention effect on both questionnaires and psychometric measures, previous studies have used change from baseline. However, most of these measures do not have a minimal clinical important difference (MCID) value which somewhat complicates clinical value. Thus, a more valuable value to consider clinical effect is using Cohen’s D effect size (see Tables), where an effect size of 0.2 is considered small, 0.5 moderate, and 0.8 large. An effect size of 0.2 can be considered as MCID^33^. In our clinical perspective, we consider an effect size of over 0.5 to be a meaningful clinically significant improvement. Thus, most of the significant statistical changes found in the HBOT compared to sham group, can be considered as clinically significant.

The significant improvement in brain microstructure (MD) induced by HBOT in the current study was in certain cortical regions which correlate with the significant changes in cognitive function including executive function, working memory and verbal fluency. These regions have been previously associated with PPCS symptoms and cognitive dysfunction in PPCS patients. The fusiform gyrus has been found to correlate with PPCS symptoms, specifically executive function, and emotional functioning^34,35^. Both the insula, inferior frontal cortex and the supramarginal gyrus have been associated with working memory^36^.

Regarding safety, the relatively high rate of adverse events (86–90% of the patients), is to be expected when treating children, as our previous work has found a higher risk of 2.7 compared to adults^37^. Moreover, children with cognitive dysfunction as well as ADHD, provide additional challenges. However, all side effects were considered mild and handled conservatively without any long term sequala. Notably, the similar rate of side effects, barotrauma in particular, in the sham group, provides validation to our sham protocol and blinding methods.

The majority of pediatric guidelines and previous studies focused on acute concussion management. Early sub symptom threshold aerobic exercise has shown efficacy and speeds recovery in the first month post injury^10,11^. However, the literature on persistent pediatrics PPCS remains limited, with few randomized controlled trials and without an effective modality. Both cognitive behavioral treatment and melatonin have been shown effective for sleep disturbance in pediatric PPCS, but did not help in behavior or cognition^38,39^. Perceptual cognitive training did not show any benefit^40^ and transcranial direct current stimulation has yet to show positive effects^41^. Collaborative care intervention, including cognitive behavioral treatment, medications and care management, have shown to reduce PPCS symptoms with a small effect size^42^. In the current study, we found that HBOT’s net effect size of global cognitive improvement was 0.598 (medium effect size) and the behavioral changes were at magnitudes of 0.5–1 (medium-large effect sizes). These effects exceed any other suggested intervention, and these effects were seen even after several years following the acute injury.

Evidence for HBOT’s effectiveness in adult with PPCS has been accumulating in the past decade. In our original adult RCT^20^, 56 civilians with PPCS 1–6 years after the acute injury were included in a crossover design study. The HBOT group, which received 40 daily sessions at 1.5 ATA, showed significant improvements in all cognitive functions: memory, executive functions, attention, and information processing speed. The control group, which received standard treatment, had no significant change in any of the parameters. Several Department of Defense (DoD) funded clinical trials followed with inconclusive findings due different methodological flaws^43–45^. Weaver et al.^23^, recently completed a trial funded by the DoD-Va, which included 71 active military servicemen and veterans who suffered from PCS more than three months to five years after mild TBI. Participants were divided into two groups: 40 daily HBOT sessions at 1.5 ATA or 1.2 ATA breathing air (which cannot be considered placebo or inert), given throughout 12 weeks. The HBOT group had significant improvements in their PPCS symptoms questionnaires as well as the cognitive processing speed and sleep measures after 13 weeks, but these results showed regression six months later. Harch et al.^24^ randomized 50 military and civilian patients suffering from PPCS following mTBI to either 40 HBOT sessions at 1.5 ATA in eight weeks or an equivalent no-treatment control period, similar to the design by Boussi-Gross et al.^20^. HBOT subjects experienced significant improvements in PPCS symptoms and cognitive function as well as depression, anxiety, sleep, and quality of life metrics. The current study is the first to evaluate the application of HBOT in pediatric PPCS. Due to our accumulated experience with PPCS patients who further benefitted from 60 sessions^21^, our pediatric protocol included 60 sessions rather than 40 sessions. Notably, standing out from the above trials, our study is the first to include a true inert sham intervention, without repeated high pressure exposures.

The therapeutic mechanisms of HBOT in PPCS have been shown in both animal models and humans. These include neuroplasticity by stimulating cell proliferation^46^, promoting neurogenesis of endogenous neural stem cells^47^, regenerating axonal white matter^48^, improving maturation and myelination of injured neural fibers^49,50^, and stimulating axonal growth, thus increasing the ability of neurons to function and communicate with each other^51,52^. This effect on neuronal microstructure has been demonstrated by our group using MRI-DTI in adults with PPCS^19^. On the cellular level, HBOT can improve cellular metabolism, reduce apoptosis, alleviate oxidative stress and restore mitochondrial function (in both neurons and glial cells)^47,53,54^. In addition to neuroplasticity, HBOT induces angiogenesis, the creation of new blood vessels. By inducing angiogenesis, HBOT improves the cerebral vascular blood flow necessary for neurogenesis and synaptogenesis^55,56^. We have demonstrated this effect on cerebral blood flow in adults with PPCS using MRI-DSC^57^.

The study has several limitations and strengths worth mentioning. First, the sample size is considerably limited and underpowered, as we were unable to recruit the needed number of eligible patients in four years. The main reason was parents refusal to have their child participate in a sham controlled study. Parents mentioned that after reading both the literature and study’s consent form containing available data on HBOT effects in adults with PPCS, they did not like the 50% chance of sham treatment. This may serve as a possible setback to any future sham-controlled study design in adult or pediatric PPCS. However, the presence of significant changes seen in a small group suggests the high potency of the intervention. Second, up to a third of patients used ADHD related drugs, which were not an exclusion criterion as long as the medications were unchanged. However, these medications can play a role in both cognitive function and behavior symptoms. Due the low number of patients, a post hoc sensitivity analysis is underpowered to draw conclusions. Third, the duration of the effect is yet to be determined in long-term follow-ups. Fourth, the current protocol included one-hour daily sessions without air breaks, similar to our previous study in adults suffering from PPCS. The use of longer daily sessions including air breaks, which were shown to be beneficial in different adult populations, remains to be determined in future studies. Along with these limitations, several strengths should be emphasized. This was a first of its kind sham double blinded controlled study, with a true inert control protocol and effective blinding of patients. Secondly, In addition to the subjective questionnaires filled by the parents, we used objective evaluations with reliable test–retest validity.

In summary, the study suggests that HBOT improves cognitive and behavioral function, PPCS, and quality of life in children suffering from PPCS even years after injury. Additional data is needed to optimize the protocol and to characterize the children who can benefit the most from this treatment.

## Methods

### Patients

Patients were 8 to 15 years old with mTBI (loss of consciousness with duration of 0–30 min, post-traumatic amnesia with duration of less than 24 h, Glasgow Coma Scale (GCS) grade of 13–15) or moderate TBI (GCS of 9–12 as well as additional criteria such as CT abnormalities) within six months to 10 years prior to the inclusion in the study), suffering from at least two post-concussive syndrome symptoms (PCS) as measured by the post-concussion symptom inventory for at least three months. Included patients had no change in cognitive or behavioral functions for at least a month prior to the beginning of the study.

Patients were excluded if they had any dynamic neurologic improvement or worsening during the past month, had been treated with HBOT for any other reason prior to their inclusion, suffered from any chest pathology incompatible with pressure changes (including asthma), inner ear disease, claustrophobia, previous neurologic conditions (e.g., epilepsy, neuromuscular diseases, metabolic diseases, etc.), brain tumors, skull base fractures, active malignancy or encephalomalacia. Patients who underwent intracranial surgery including ventricular drainage, subdural hematomas drainage, epidural hematomas drainage and intracerebral hemorrhage evacuation were excluded as well. Lastly, patients who were unable to perform awake brain MRIs were excluded.

### Screening procedure

After signing the informed consent (by the parents), patients underwent screening cognitive assessments, where children over the age of ten underwent a computerized battery (Neurotrax, see below) and children younger than 10 underwent pen and paper based tests. Patients were included in the study with a decrease of at least one standard deviation (SD) in at least one of the following domains: memory, attention, information processing speed, executive function than expected per age and education level.

### Trial design

A prospective randomized, double blind, sham-controlled trial was conducted from December 1st, 2017, to November 1st, 2021, at Shamir Medical Center (SMC), Israel. Eligible patients were randomized to either HBOT or sham-control groups in a 1:1 ratio according to a computerized randomization table, supervised by a blinded researcher. To evaluate participant masking, patients were questioned after the first session on their perception regarding the treatment they received. An evaluation procedure was done at baseline and 1–3 weeks after the last HBOT or sham session. All evaluators were blinded to the patients’ group allocation. The study was approved by SMC’s Institutional Review Board (IRB) (No. 120-16-ASF) and all participants’ parents signed an informed consent prior to their inclusion. All research was performed according to the relevant guidelines and regulations. This study was registered with ClinicalTrials.gov, number NCT03339037 on 13/11/2017.

### Intervention

Both HBOT and sham protocols were administrated in a multi-place Starmed-2700 chamber (HAUX, Germany). The protocol comprised of 60 one-day sessions, five sessions per week within a three-month period. The HBOT protocol included breathing 100% oxygen by mask at 1.5 ATA for 60 min with no air breaks. Compression/decompression rates were 1.0 m/minute. The sham protocol included breathing 21% oxygen by mask at 1.03 ATA for 60 min. To mask the controls, the chamber pressure was raised up to 1.1 ATA during the first five minutes of the session along with circulating air noise followed by decompression (0.4 m/minute) to 1.03 ATA during the next five minutes.

### Outcome measures

#### Cognitive function

- Computerized cognitive health assessments were evaluated by the NeuroTrax computerized cognitive testing battery (NeuroTrax Corporation, Bellaire, TX)^58,59^. This assessment comprises of several cognitive tests that evaluate various aspects of brain capabilities including memory, executive function, attention, information processing speed, and motor skills. The cognitive indices were based on scores of six cognitive tests: verbal memory, non-verbal memory, go–no-go test, Stroop test, staged information processing test and the catch game. The cognitive domains are a combination the several sub-tests, where the global score is the mean value of the cognitive domains. Outcome parameters were calculated using custom software, blind to diagnosis or the testing site^59^. Domain scores were normalized for age and gender. Additional information is also available on the NeuroTrax website (http://www.neurotrax.com/).
- Trail making test: The trail making test children’s version, parts A and B^60^, measures mental set-shifting, attention, and cognitive processing speed. The test can provide information about visual search speed, scanning, speed of processing, mental flexibility, as well as executive functioning. The task requires a subject to connect a sequence of 25 consecutive targets on a sheet of paper. There are two parts to the test. In the first part, the targets are all numbers from 1 to 25 and the test taker needs to connect them in sequential order. In the second part, the dots go from 1 to 13 and include letters from A to L (in Hebrew script, Aleph to Lamed). As in the first part, the patient must connect the dots in order while alternating letters and numbers in the shortest time possible without lifting the pen from the paper. Scoring is based on time taken to complete the test with lower scores being better.
- The Wechsler Intelligence Scale for Children-4th Edition (WISC-IV Integrated): The WISC–IV^61^ is a psychometric measure of intelligence in children who are between 6 and 16 years of age. We performed five subsets out of the 10: the WMI which included the digit span and letter–number sequencing subtests, the PSI which includes the coding and symbol search subtests and the cancellation. The digit span and coding subtests are reliable and valid measures of short-term memory/working memory and speed of information processing in children and adults. The digit span subtest measures the participant's ability to repeat numbers read aloud by the examiner in a forward and backward order. The coding subtest requires the participant to reproduce symbols in a sequence as quickly as possible.
- Test of memory and learning (TOMAL): The TOMAL^62^ is a memory battery that is designed for children 5 to 19 years old. It is composed of 10 core subtests (memory for stories, word selective reminding, object recall, digits forward, paired recall, facial memory, visual selective reminding, abstract visual memory, visual sequential memory, and memory for location), and four supplemental subtests (letters forward, digits backward, letters backward, and manual imitation). The core subtests combine to produce a number of composite indices, and the verbal memory index, nonverbal memory index, and attention/concentration index were used in this study.
- The Rey auditory verbal learning test (RAVLT): The RAVLT is a neuropsychological assessment designed to evaluate verbal memory in patients^63^. Five presentations of a 15-word list are given, each followed by an attempted recall. This is followed by a second 15-word list (list B), followed by recalling of list A. Delayed recall and recognition are also tested. A key feature of the RAVLT is that it affords the opportunity to measure rates of learning, as opposed to recall of a single stimulus, or series of stimuli.
- The five points test (5PT): The 5PT^64^ is a structured and standardized test that assesses figural fluency functions which are associated with executive functioning. A participant is asked to generate as many unique designs as possible in a certain time limit. The number of completed correct designs are counted after one and two minutes have elapsed.
- Verbal fluency: Verbal fluency is a cognitive function that facilitates information retrieval from memory. Successful retrieval requires executive control over cognitive process such as selective attention, mental set shifting, internal response generation, and self-monitoring. The FAS test^65^ measures both semantic and phonemic word fluency. It assesses phonemic fluency by requesting an individual to orally produce as many words as possible that begin with specific letters within a prescribed time frame, usually one minute. The semantic fluency test requires a subject to generate as many words as possible from a given semantic category (e.g., “fruits”) within a limited time, usually one minute.
- The NEPSY, Second Edition (NEPSY-II): The NEPSY-II is a standardized neuropsychological battery for children aged 3–16 years that assesses functioning across six domains: executive functioning and attention, memory and learning, sensorimotor functioning, social perception, language, and visuospatial processing^66^. The NEPSY-II yields both raw and standardized scores (by age) and has adequate-to-high reliability (including internal consistency and test-retest reliability), validity, and interscorer agreement. For the purpose of this study, we administered the animal sorting subtest from the NEPSY-II battery used to test the domains of attention and executive functioning. Each subtest used in this study includes a teaching example or learning trial in which the examiner is able to correct the child’s errors and to ensure that the child understands the task. The subsets yield a mean standard score of 10 with a standard deviation of three.

#### Post-concussion symptoms

- The British Columbia post-concussion symptom inventory (BC-PSI): The BC-PSI is a 16-item measure designed to assess the presence and severity of PCS symptoms. Parents were asked to rate the frequency and intensity of 13 symptoms over the past two weeks, including that day. The symptoms included items such as headache, poor sleep, temper problems, and poor concentration. The psychological symptoms were rated on a Likert-type scale measuring frequency from 0 (not at all) to 5 (constantly), and the intensity ratings ranged from 0 (not at all) to 5 (very severe problem)^67^. Scoring was calculated by multiplying frequency by intensity to create a single score for each item, and this score was then converted to a score on a scale of 0–4. The produced score of 0–1 was considered: 0: 2–3; 1: 4–6; 2: 8–12; 3: 15+ —4. These scores represent severity whereby 0—none, 1–2—mild, and 3 +—moderate or greater. Similarly, total score representations are 0 = low, 1–9 = normal, 10–14 = unusually high, and 15 +  = extremely high, relative to healthy adults. The internal consistency reliability for the items comprising the total score is r = 0.82^67,68^.
- Health and behaviour inventory (HBI): The HBI is a 20-item scale that measures the frequency of different symptoms that are associated with concussion^69^. Parents completed the child version of the HBI by rating the frequency of symptoms experienced on a 4-point Likert scale as 0 (never), 1 (rarely), 2 (sometimes), and 3 (often). In addition to individual symptom ratings, a total symptom score was calculated as the sum of all items in which higher scores indicate more frequent symptoms.

#### Quality of life

- Paediatric quality of life inventory (PEDSQL): The PedsQL-4.0 is a reliable and valid measure of health related quality of life (HRQoL) in healthy children and those with acute and/or chronic health conditions^70^. Parents were asked to complete the parents versions which exist for children aged 2–18 years (in four age groups). Items are calculated and transformed into an overall score with a range of 0 to 100 points, with more points indicating better HRQoL. Secondary outcomes included the PedsQL-4.0 subscale scores: physical, emotional, social, and school. The MCID is estimated at 4.88-6.27^71^.

#### Behavioural assessment

- Conner's Rating Scale: The Conner's Rating Scale is a reliable and valid parent questionnaire assessing their child's functioning in emotional, social, cognitive and behavioral domains. The emphasis of this measure is attentional in nature^72^ and is useful in monitoring change in behavior across the day^73^. The Conner’s rating scale contains four component subscales: ADHD index, hyperactivity, cognition, and appositional. The ADHD index subscale contains 12 items, three of which are also used in the 6-item cognition problems/inattentive subscale^74^. The hyperactivity, cognition, and appositional subscales each contain six items. The ADHD index subscale provides an indicator of children likely to have attentional problems. The hyperactivity scale assesses both the hyperactivity and impulsivity domains of ADHD symptoms. The appositional subscale assesses appositional behaviours. The cognition problems/inattention subscale assesses traits that may lead to difficulties in school academic performance^74^. Compared with clinicians and teachers, parents have more opportunities throughout the day to observe the impact of ADHD on quality of life, during activities that are school related (such as homework) and non-school-related (such as chores or play)^74^.
- Behavioural rating inventory of executive function (BRIEF): BRIEF is a reliable and valid parent questionnaire assessing their child's executive functioning^75^. The questionnaire was designed for parents of school-aged children that enables professionals to assess executive function behaviours at home. It contains 86 items within eight theoretically and empirically derived clinical scales that measure the different aspects of EF: inhibition, shift, emotional control, initiate, working memory, planning/organization, organization of materials, and monitor^75^.

#### Neuro-physical evaluation

- The balance error scoring system (BESS) is a useful means of measuring postural stability, which frequently exhibits deficits after a concussion^76^. Patients were tested in three positions (feet touching side-by-side, a single leg stance on the nondominant leg, and a heel-to-toe stance with the dominant foot in front) for 20 s each, on a firm surface and then on a 50 cm × 41 cm × 6 cm–thick foam pad. The dominant leg was identified by asking subjects which foot they would use to kick a ball. Patients were instructed to close their eyes and place hands on hips for 20 s. Subjects were also told that the clock would not stop running during testing, so if they ever came out of the starting position, they should make any necessary adjustments and return to the position as quickly as possible. One error point was given every time the subject moved hands off hips, opened eyes, stepped, stumbled, abducted or flexed the hip greater than 30°, lifted the forefoot or heel from the testing surface, or remained out of the testing position for more than five seconds. The maximum score for any one trial was 10 points. If a subject could not maintain one of the positions for a minimum of five seconds at any point during a trial, we assigned the maximum score of 10 for that trial. We calculated the total BESS score (sum of scores from all six trials), the firm surface score (sum of scores from the three firm surface trials), and foam surface score (sum of scores from the three foam surface trials).

#### Brain imaging

- Brain imaging MRI scans were performed on a MAGNETOM Skyra 3 T scanner, configured with 20-channel receiver head coils (Siemens Healthcare, Erlangen, Germany). The MRI protocol included T2-weighted, 3D fluid attenuated inversion recovery (FLAIR), susceptibility weighted imaging (SWI), pre- and post-contrast high-resolution MPRAGE 3D T1-weighted, and diffusion tensor imaging (DTI).
- Whole brain diffusion weighted images were acquired with the following parameters: 50 axial slices, slice thickness = 2.2 mm, voxel size = 1.5 × 1.5 mm, TR = 9300 ms, TE = 91 ms, and matrix = 128 × 128 mm. Diffusion gradients were applied along 30 noncollinear directions (b = 1000 s/mm2), and one volume without diffusion weighting.
- Preprocessing of DTI images was preformed using the SPM software (version 12, UCL, London, UK) and included motion correction, co-registration with MPRAGE T1 images, spatial normalization, and spatial smoothing with kernel size of 6 mm full width half maximum (FWHM). Diffusion brain volume denoising was performed using the joint anisotropic LMMSE filter for stationary rician noise removal^77^ and calculation of DTI-FA (fractional anisotropy) and MD (mean diffusivity) maps were performed using in house software written in MATLAB R2021b (MathWorks, Natick, MA).

#### Safety

Participants were monitored for adverse events including barotraumas (either ear or sinuses), and oxygen toxicity (pulmonary and central nervous system).

### Statistical analysis

Continuous data are expressed as means ± standard deviations (SD). Two-tailed independent t-tests were performed to compare variables between groups when a normality assumption held according to a Kolmogorov–Smirnov test. Net effect sizes were evaluated using Cohen's d method, defined as the improvement from baseline after sham intervention was subtracted from the improvement after HBOT, divided by the pooled standard deviation of the composite score. Categorical data are expressed in numbers and percentages, compared by chi-square/Fisher’s exact tests. A value of p < 0.05 was considered significant.

Imaging data analysis was performed on the normalized FA and MD maps, using the voxel-based method to generate statistical parametric maps. A gray matter mask was applied on the MD maps, and a white matter mask on the FA maps (using a threshold of 0.2). A within-subject repeated measure ANOVA model was used to test the main interaction effect between time and group implemented in SPM software (version 12, UCL, London, UK). Correlations between MD changes and cognitive scores were performed using Pearson’s correlation. Data analysis was performed using MATLAB R2021b (MathWorks, Natick, MA) Statistics Toolbox.

#### Sample size

The estimated sample size was calculated based on test reliability in healthy volunteers^78^. A NeuroTrax global cognitive score improvement of 2% in the placebo and 12% in the treatment arm, with a mean score of 100 and standard deviation of 14, to demonstrate a 10% effect. Assuming a power of 80%, and 5% two-sided level of significance, a total of 62 participants would be required, 31 participants in each arm. Considering a dropout rate of 15% the total sample size required is 70.

## Data Availability

The datasets analyzed during the current study are available from the corresponding author on reasonable request.
